# The Impact of Cardiac Telerehabilitation on Health-Related Quality of Life in Patients Undergoing Percutaneous Coronary Intervention (PCI): A Systematic Review Protocol

**DOI:** 10.3390/nursrep14040291

**Published:** 2024-12-13

**Authors:** Francesco Limonti, Andrea Gigliotti, Francesco Gravante, Nicola Ramacciati

**Affiliations:** 1 Department of Biomedicine and Prevention, University of Rome Tor Vergata, 00133 Rome, Italy; 2 Department of Health and Exercise Science, The University of Oklahoma, Norman, OK 73019, USA; andrea92cv@gmail.com; 3 Department of Critical Care, Local Health Authority of Caserta, San Giuseppe Moscati Hospital, 81031 Aversa, Italy; francesco.gravante@aslcaserta.it; 4 Department of Pharmacy, Health and Nutritional Sciences (DFSSN), University of Calabria, 87036 Rende, Italy; nicola.ramacciati@unical.it

**Keywords:** cardiac telerehabilitation, percutaneous coronary intervention, health-related quality of life, systematic review, percutaneous angioplasty

## Abstract

Background: Cardiac rehabilitation (CR) is an intervention to improve health and quality of life in patients undergoing percutaneous coronary intervention (PCI). The use of digital technology for healthcare promotion, such as telemedicine, has received growing attention in recent years due to the possibility of offering remote and individualized cardiac rehabilitation to patients undergoing coronary interventions. However, the impact of cardiac telerehabilitation on health-related quality of life (HRQoL) is not fully understood. This systematic review aims to analyze through meta-analyses and synthesized comments the current knowledge on the effectiveness of cardiac telerehabilitation in improving HRQoL in patients undergoing PCI. Objectives: This manuscript presents a protocol for a systematic review to assess the effects of cardiac telerehabilitation on HRQoL in cardiac patients after PCI. Furthermore, the systematic review will explore the different modalities of remote rehabilitation documented in scientific literature. Methods: The literature review protocol was developed according to the PRISMA guidelines for systematic reviews. Search terms were structured according to the PIO (Population-Intervention-Outcome) framework. All relevant available studies will be identified using the main databases (PubMed, Scopus, CINAHL, Web of Science, and the Cochrane Library) and included in the review. Methodological quality and risk of bias will be evaluated using the Crowe Critical Appraisal Tool (CCAT). This review protocol has been registered on PROSPERO (No. CRD42024582933). Conclusions: This systematic review will comprehensively investigate the effects of cardiac telerehabilitation on QoL improvements in patients after PCI.

## 1. Introduction

Cardiovascular diseases (CVDs) are one of the leading causes of morbidity and mortality worldwide [1]. Furthermore, CVD negatively impacts patients’ quality of life, and it represents a burden for the national healthcare system [1]. Despite advancements in medicine and healthcare, the number of CVD-related deaths, as estimated by the World Health Organization (WHO), was 4.2 million in 2019 in Europe [2]. Currently, 42.5% of total deaths in Europe are attributable to CVD [2]. Coronary disease is one of the most severe CVDs, especially in developed countries, where primary and secondary prevention significantly improves patient lifespan [3].

In recent decades, scientific and clinical advances have led to the development of new effective techniques to treat CVD, such as percutaneous coronary intervention (PCI) [4]. PCI is a minimally invasive and more accessible intervention to treat myocardial infarction (MI) and represents an alternative to traditional surgery. However, the long-term success of this intervention also depends on the efficacy of post-surgery cardiac rehabilitation (CR) [5].

According to the WHO, CR is a multidisciplinary intervention to optimize the physical, psychological, and social conditions of patients affected by CVD [6]. Moreover, CR has effectively improved clinical conditions and health-related quality of life (HRQoL) in patients post-MI [7]. Furthermore, CR reduces the risk of new MI events and consequent hospitalization [8].

Traditional rehabilitation programs present limitations that negatively impact patients’ accessibility to the rehabilitation sessions [8]. For instance, patients could live in a geographic area where CR programs are unavailable or experience difficulty organizing their schedules to attend rehabilitation sessions [9,10]. Remote rehabilitation could represent a solution to reduce some of the above-mentioned limitations [11]. Telemedicine and telenursing are flexible and personalized approaches that allow patients to receive constant and professional home-based rehabilitation [12,13]. Few studies have explored the long-term outcomes of telerehabilitation in HRQoL and its potential to replace or complement traditional rehabilitation models [14,15]. More research is required to better understand the efficacy of home cardiac rehabilitation programs across different populations and health systems. These programs have the potential to improve adherence and achieve results comparable to, or even superior to, traditional rehabilitation approaches [11].

Nursing plays a key role in managing and conducting telerehabilitation programs [16]. Furthermore, telenursing has been shown to positively impact cardiac patients post-MI by offering constant adjustments to the rehabilitation program and consequently reducing risk factors such as new MI events [14,17].

Remote cardiac rehabilitation led by nurses is an area of growing interest due to its potential to increase rehabilitation program adherence and reduce geographic and time availability barriers that limit the effectiveness of traditional rehabilitation interventions [16].

Considering the importance of these interventions, it is crucial to systematically analyze the evidence related to the effectiveness of cardiac telerehabilitation programs on quality of life, clinical outcomes, and the risk of new MI events.

### Aim

We aim to develop a protocol for a systematic review focusing on the effects of home-based CR on HRQoL in cardiac patients who have undergone PCI.

Our research question is: can cardiac telerehabilitation improve HRQoL and reduce new cardiac events in patients post-PCI?

We hypothesize that home-based cardiac rehabilitation, particularly when mediated by telenursing, will lead to significant improvements in HRQoL compared to traditional rehabilitation approaches. Additionally, we hypothesize that telerehabilitation will reduce the incidence of other ischemic cardiac events, such as MI, due to enhanced patient adherence and individualized care.

## 2. Materials and Methods

### 2.1. Eligibility Criteria

A preliminary review protocol was developed to ensure that the selected articles match the objectives of the systematic review. The results are presented to provide a clear and useful message to the scientific community. The systematic review will follow the PRISMA guidelines. Specifically, the PRISMA-P guidelines [18,19] have been used to develop the review protocol [see the PRISMA checklist in the Supplementary Files], while the PRISMA guidelines will be used for the review manuscript [20].

#### 2.1.1. Information Sources

The review will include primary studies, such as randomized controlled trials, cohort studies, cross-sectional studies, case–control studies that answer our research questions, and qualitative studies. This review protocol used the PIO (Population-Intervention-Outcome) framework to provide a robust design that can specifically answer our research question. To conduct a thorough review, carefully crafted search terms will be identified and applied across multiple databases. This approach will enable an extensive exploration of resources, including MEDLINE (via PubMed), CINAHL (via EBSCO), the Cochrane Library (via Embase), Web of Science, and Scopus. All relevant studies up to the date of extraction will be included. Based on the relevance of our research question, the PIO framework will allow us to demarcate the study area (Table 1). Table 2 shows the key terms used to perform the search. Subsequently, we will utilize the software Rayyan (Rayyan Enterprise, Cambridge, MA, USA, https://www.rayyan.ai/ (access date 27 October 2024)) to eliminate duplicates [21]. Two authors will independently select the articles to include in the review and a third author will resolve potential conflicts. Full texts will be read once the articles are selected, and a final decision will be made on their inclusion or exclusion.

#### 2.1.2. Search Strategy

The systematic search was developed following the PIO framework described in Table 1.

### 2.2. Inclusion and Exclusion Criteria

The inclusion criteria for this systematic review will include a broad range of relevant studies. All studies published in peer-reviewed journals that answer our research questions will be eligible for consideration. Only studies that include patients undergoing remote cardiac rehabilitation will be included. Only studies published in English will be included in this review. This decision will be made to ensure consistency in data interpretation, address resource constraints related to translation, and focus on the primary language of high-impact academic publications in the field.

The exclusion criteria will outline the parameters to maintain the focus and integrity of the review. Unpublished studies will be excluded, including conference abstracts, theses, and study protocols. Similarly, editorial pieces and opinion articles lacking primary data, animal studies, and experimental or laboratory models will be excluded from the scope of this review. Additionally, studies focusing exclusively on infant and pediatric populations (<18 years) will be excluded from consideration. Unpublished studies and theses generally lack the rigorous evaluation of peer review, raising concerns about their reliability and scientific validity. Conference abstracts, while useful for initial insights, are typically concise and fail to provide the depth of analysis or comprehensive data needed for robust conclusions. Trial protocols, on the other hand, outline planned methodologies but do not offer actual results or evidence, limiting their relevance for evidence-based assessments. We recognize that excluding non-English studies and unpublished or gray literature may introduce some level of language and publication bias. However, this approach will be adopted to prioritize methodological rigor and feasibility. These criteria will be carefully established to ensure that the review will include a full range of relevant studies.

### 2.3. Quality Appraisal and Risk of Bias

The comprehensive assessment will include validated risk of bias and quality assessment tools using the Crowe Critical Appraisal Tool (CCAT) to provide a detailed examination of the studies [22]. The CCAT serves as an instrument capable of assessing the methodological quality across a diverse array of research designs, including quasi-experimental, descriptive-exploratory-observational, qualitative, systematic review, and true experimental designs. CCAT consists of 22 criteria divided into 8 main sections: Preliminaries, Introduction, Design, Sampling, Data Collection, Ethical Matters, Results, and Discussion, providing a comprehensive framework for critical appraisal [23]. The evaluation process will be independently carried out by two researchers to minimize the risk of subjective bias. Any discrepancies in their assessments will be thoroughly discussed to reach a consensus. If disagreements cannot be resolved, a third independent reviewer will provide an impartial evaluation to ensure a conclusive resolution.

The CCAT scoring system reflects the methodological quality of each study, with individual categories rated on a scale from 0 (low quality) to 5 (excellent quality). The overall score will be calculated by summing the values obtained across the eight sections, allowing for a global classification of study quality. Studies with scores below a predefined threshold, indicative of inadequate methodological quality, will be excluded from the final analysis, in agreement with the PRISMA guidelines. The rigorous application of the CCAT identifies potential biases in individual studies, including selection, measurement, and friction biases, clarifying methodological limitations. The main sources of bias, such as lack of randomization, non-representative samples, or non-standardized data collection methods, will be analyzed and reported to ensure transparency and reliability of the results. To further enhance the rigor of the analysis, the ROBINS-I tool will also be incorporated to establish clear cut-off thresholds to classify the risk of bias as high, medium, or low. This will allow for a more structured and transparent assessment of bias levels, complementing the full assessment provided by the CCAT framework.

### 2.4. Study Records

The manuscript will adhere to the Preferred Reporting Items for Systematic Reviews and Meta-Analyses Protocols (PRISMA) guidelines [16]. Data extraction and analysis will be conducted in two stages. Initially, two authors will independently examine the titles and abstracts of the retrieved literature using the Rayyan software [21] to identify potential articles for inclusion in the systematic review. Potential discrepancies will be resolved through discussion between the authors and the involvement of a third author if conflicts persist. Subsequently, the same two authors will independently read the full texts of the selected articles and perform data extraction. Any inconsistencies in the abstracted data will be addressed through discussion between the authors with the involvement of a third party, if necessary. To ensure high quality and reliability in the systematic review, we will implement a rigorous evaluation process. This process will thoroughly assess each included study’s methodological robustness and relevance. Two independent researchers will extract the variables as follows:

- Author(s)
- Year and country of study
- Type of Study
- Population
- Setting
- Intervention(s)
- Primary and secondary outcome(s)
- Results

### 2.5. Data Collection

To ensure a structured and transparent approach to the systematic review process, this review will follow the conceptual framework outlined by Kairy, D. et al. [24]. This framework provides a robust guide for synthesizing evidence across studies, allowing for a comprehensive evaluation of the impact of telerehabilitation on health-related quality of life. Furthermore, a PRISMA-based flow diagram will be included to visually summarize the review process, including the selection and exclusion of studies.

This review will adopt a systematic approach to data extraction, utilizing a standardized data extraction tool. Relevant data, including study design, participant demographics, interventions, and outcome measures, will be collected from each included study. For quantitative data, results will be synthesized through a narrative summary. For qualitative data, thematic synthesis will be employed to identify recurring patterns and insights. Studies will be categorized based on their respective levels of evidence, facilitating a comparative assessment of the strength and reliability of their findings. This mixed-methods synthesis approach will integrate qualitative and quantitative results to provide a comprehensive understanding of the impact of home-based telerehabilitation programs.

### 2.6. Data Synthesis

The key findings from each study will be presented and compared through a synoptic table. Subsequently, these findings will be critically analyzed and discussed within the context of their respective levels of evidence, allowing for a comprehensive synthesis of the cumulative evidence. As part of the analysis, we plan to report adherence rates to telemedicine to further evaluate its impact. The findings of the studies will be summarized in a narrative form, focusing on identifying patterns and themes in the data. The summary will also be used to identify areas for further research. To assess the strength of the results in the reviewed articles, the hierarchy of evidence proposed by Polit and Beck will be adopted [25]. Quantitative data, where available, will be presented descriptively, while qualitative findings will be analyzed thematically to highlight recurring insights. The narrative synthesis will follow a structured framework, including exploring relationships within and between studies and mapping findings to identify key themes and patterns. Data from the included studies will then be grouped based on the type of intervention, the characteristics of the population, and the outcomes measured.

If sufficiently homogeneous data becomes available, we will attempt to perform a meta-analysis using the RevMan 5.3 software. For the meta-analysis, we plan to use a random-effects model to account for potential variability between studies, including differences in study design, intervention types, and populations. Heterogeneity will be assessed using the I^2^ statistic, with thresholds of 25%, 50%, and 75% indicating low, moderate, and high heterogeneity, respectively. Sensitivity analyses will also be conducted to evaluate the robustness of the pooled results. Combining a structured narrative synthesis with a potential meta-analysis, this systematic review aims to provide a comprehensive and transparent evaluation of the available evidence, while addressing the variability inherent in the included studies.

### 2.7. Measures and Outcomes

To ensure a comprehensive evaluation of the impact of telerehabilitation, this review will consider various measures and outcomes. The primary outcome of interest is health-related quality of life (HRQoL), which will be assessed using validated tools such as SF-36, EQ-5D, or other recognized scales reported in the included studies. Secondary outcomes will include rates of adherence to telerehabilitation programs, incidence of recurrent cardiac events, and patient satisfaction.

If sufficient data are available, subgroup analyses will be performed to explore differences in outcomes based on patient characteristics such as age, gender, and comorbidities. These analyses will help identify how these factors influence the effectiveness of telerehabilitation, providing critical insights for tailoring interventions to specific populations. Additionally, sensitivity analyses will be conducted during the meta-analysis to assess the robustness of the findings and the impact of heterogeneity between studies.

## 3. Impact of the Review

This systematic review will include original studies conducted in cardiac patients (≥18 years of age), post-PCI, who have undergone home-based cardiac telerehabilitation. Furthermore, the impact of these rehabilitation programs on HRQoL will be evaluated. If remote cardiac rehabilitation effectively improves HRQoL, it could represent a relatively inexpensive and highly accessible intervention strategy to improve overall healthcare for cardiac patients.

## 4. Discussion—Outcome and Prioritization

The primary outcome of this systematic review is health-related quality of life (HRQoL) in patients undergoing home-based cardiac telerehabilitation post-percutaneous coronary intervention (PCI). HRQoL is assessed using validated instruments such as the SF-36, EQ-5D, or other recognized scales. Secondary outcomes include adherence to rehabilitation programs, the incidence of recurrent cardiac events, and patient satisfaction with telerehabilitation. Prioritization of outcomes are guided by their relevance to clinical practice and impact on patient-centered care. HRQoL was chosen as the primary focus due to its comprehensive reflection of the physical, psychological, and social well-being of patients [26]. This systematic review aims to evaluate the impact of home-based cardiac rehabilitation (CR), specifically telerehabilitation, on HRQoL in patients undergoing PCI. Telerehabilitation programs offer several advantages over traditional center-based programs, including greater accessibility for patients in remote areas and increased flexibility for families [27]. These programs may also lead to superior outcomes, such as improved risk factor management and reduce recurrent cardiac events [28]. However, potential challenges associated with implementing telerehabilitation programs include the reliance on technology, which may exclude certain patient populations, such as those with limited digital literacy or access to necessary equipment [29]. The findings of this systematic review will help highlight how educational interventions led by nurses and other healthcare professionals can be effectively integrated into telehealth-mediated rehabilitation care. This integration underscores the critical role of nurse-led interventions in improving patient outcomes and addressing barriers to equitable care delivery in cardiac rehabilitation.

### Implications for Clinical Practice

Telerehabilitation offers significant potential to improve accessibility and continuity of care, particularly for patients in disadvantaged or rural areas or those unable to easily reach hospitals. Technology enables healthcare professionals to provide personalized, multidisciplinary interventions that enhance health-related quality of life.

Integrating telerehabilitation into clinical practice requires addressing several structural and organizational factors, such as ensuring leadership support from healthcare workers, integrating telerehabilitation programs with existing clinical workflows, and providing adequate training for telerehabilitation providers, healthcare workers, and patients [30]. The review will explore patient engagement and adherence rates, as these factors are critical to the success of telerehabilitation programs and their long-term sustainability, providing strategies for integrating telerehabilitation into healthcare systems as a standard procedure.

## 5. Limitations

The inclusion of English-only studies may introduce language bias, potentially excluding relevant evidence published in other languages. Furthermore, the heterogeneity of telerehabilitation protocols and outcome measures across the included studies may limit the comparability of findings and the ability to draw generalized conclusions. Finally, while the use of digital technologies enhances the accessibility of rehabilitation programs, it may also exclude populations with limited digital literacy or access to necessary devices.

## 6. Meta-Bias

This systematic review will consider potential meta-biases, including the tendency to favor studies with positive outcomes and language bias due to the inclusion of English-only studies. Additionally, selection bias and heterogeneity in telerehabilitation protocols could influence the results. However, a highly sensitive method will be employed to select and critically analyze the included articles, ensuring an accurate and thorough evaluation of the available evidence.

## 7. Ethical Considerations

This systematic review will adhere to the ethical standards for the proper handling of data. All data extracted from the included studies will be handled using the principles outlined in the Declaration of Helsinki. Furthermore, the review will comply with data privacy. The studies that will be included in this review are assumed to have obtained the necessary ethical approvals and informed consent from their respective participants, as required by their institutional guidelines. This approach ensures transparency, accountability, and respect for ethical standards throughout the review process.

## Figures and Tables

**Table 1:** PIO framework.

PICO Model	Description
Population (P)	The population included in this review is composed of individuals ≥ 18 years of age who have undergone PCI after MI.
Intervention (I)	The intervention includes programs of cardiac rehabilitation conducted remotely via telemedicine or telenursing. These programs aim to provide higher flexibility, individualization, and better adherence to the rehabilitation process as they allow the patients to comfortably follow the sessions from their own homes.
Outcome (O)	The primary outcome of this review is health-related quality of life (HRQoL) post-rehabilitation, measured using validated tools reported in the included studies. Studies will be considered eligible if HRQoL is assessed quantitatively through established measurement instruments, such as SF-36, EQ-5D, or other validated scales. Interventions included will involve home-based telemedicine programs, and participants may require access to devices such as phones, tablets, or computers to engage in the rehabilitation process.

PCI: percutaneous coronary intervention; MI: myocardial infarction; HRQoL: health-related quality of Life; CR: cardiac rehabilitation.

**Table 2:** Key terms and search strategy.

Population	Intervention	Outcome
angioplasty coronary intervention OR angioplasty coronary revascularization OR percutaneous coronary intervention OR percutaneous OR angioplast * OR endoluminal repair OR stent * OR acute coronary syndrome * OR Percutaneous coronary angioplasty OR myocardial infraction OR myocardial ischemia OR coronary artery disease OR acute coronary syndrome OR ischemic heart disease OR heart disease * OR heart attack OR coronary heart disease OR coronary disease * OR acute coronary syndrome * OR angina * OR percutaneous coronary revascularization * OR percutaneous transluminal coronary angioplast * OR coronary balloon angioplast * OR percutaneous endoluminal repair	Home-based rehabilitation OR telenursing OR telemedicine OR telerehabilitation OR remote rehabilitation OR e-health OR telehealth OR internet-based OR home-based rehabilitation OR home-based cardiac telerehabilitation OR home-based cardiovascular rehabilitation OR home-based exercise therapy OR home-based exercise training OR Mhealth OR m-health OR e-health OR mobile health OR digital health OR mobile application OR mobile device OR mobile communication * OR mobile phone OR smartphone OR smart phone OR smartphone application OR tele-rehabilitation OR virtual rehabilitation OR remote rehabilitation OR telehealth OR tele-health OR telemonitor *	Quality of life OR health-related quality of life OR HRQOL

Each concept was be combined with AND; the * symbol is used to explode key search terms in MEDLINE.

## Data Availability

No new data were created or analyzed in this study. Data sharing is not applicable to this article.

## Supplementary Materials

The following supporting information can be downloaded at: https://www.mdpi.com/article/10.3390/nursrep14040291/s1, Table S1: PRISMA-P (Preferred Reporting Items for Systematic review and Meta-Analysis Protocols) 2015 checklist: recommended items to address in a systematic review protocol *.

## Public Involvement Statement

There was no public involvement in any aspect of this research.

## Guidelines and Standards Statement

In writing this manuscript we followed the PRISMA-P 2015 Checklist guidelines.
